# Research progress in the role and mechanism of Cadherin-11 in different diseases

**DOI:** 10.7150/jca.52720

**Published:** 2021-01-01

**Authors:** Xinyi Chen, Hongjiao Xiang, Shiyu Yu, Yifei Lu, Tao Wu

**Affiliations:** Institute of Interdisciplinary Integrative Medicine Research, Shanghai University of Traditional Chinese Medicine, Shanghai 201203, China.

**Keywords:** cadherin-11, epithelial-mesenchymal transition, transforming growth factor

## Abstract

Cadherin is an important cell-cell adhesion molecule, which mediates intercellular adhesion through calcium dependent affinity interaction. Cadherin-11 (CDH11, OB-cadherin) is a member of cadherin family, and its gene is situated on chromosome 16q22.1. Increasing lines of researches have proved that CDH11 plays important roles in the occurrence and development of a lot of diseases, such as tumors, arthritis and so on. CDH11 often leads to promoter methylation inactivation, which can induce cancer cell apoptosis, suppress cell motility and invasion, and can inhibit cancer through Wnt/β-catenin, AKT/Rho A and NF-κB signaling pathways. This review focused on the current knowledge of CDH11, including its function and mechanism in different diseases. In this article, we aimed to have a more comprehensive and in-depth understanding of CDH11 and to provide new ideas for the treatment of some diseases.

## Introduction

Homotypic cadherin interactions mediated the cell-cell adhesion. Cadherins can gather to form homodimers by a zipper-like mechanism, and the intracellular domain is anchored to the actin cytoskeleton by α-catenin and β-catenin. Cadherins are transmembrane Ca^2+^-dependent homophilic adhesion receptors, which are vital for cellular processes, including the proliferation, differentiation and migration of cyto-skeletal organizations, and for maintenance of structural integrity of tissues and homeostasis 1. They also play important roles in recognition and sorting of cells during development 2. Six-cadherin cluster, including Cadherin-1 (CDH1), Cadherin-3 (CDH3), Cadherin-5 (CDH5), Cadherin-8 (CDH8), Cadherin-11 (CDH11) and Cadherin-13 (CDH13), are located on chromo-some 16 (16q22.1-16q24.3). In previous studies, CDH1 and CDH13 were proved as functional tumor inhibitory factors, involved in inhibiting the invasion, proliferation and metastasis of tumor cells 3. Cadherins are divided into type I and type II, and CDH11 is a latter type, which is broadly expressed in mesenchymal stem cells (MSCs), smooth muscle cells (SMCs), fibroblasts and osteoblasts. CDH11 expression is very important during epithelial mesenchymal transition (EMT). The proliferation and survival of some cells, including vascular SMCs, osteosarcoma and glioblastoma, are also strongly correlated with the expression of CDH11 3,4. More and more studies have showed that CDH11 plays important roles in the process of tumor, metabolic diseases, arthritis, skin diseases and other diseases, and may be a potential therapeutic target. Therefore, this article reviews the role and mechanism of CDH11 in the development of various diseases in order to further understand it from a macro perspective.

## Structure and function of CDH11

**Figure 1:**
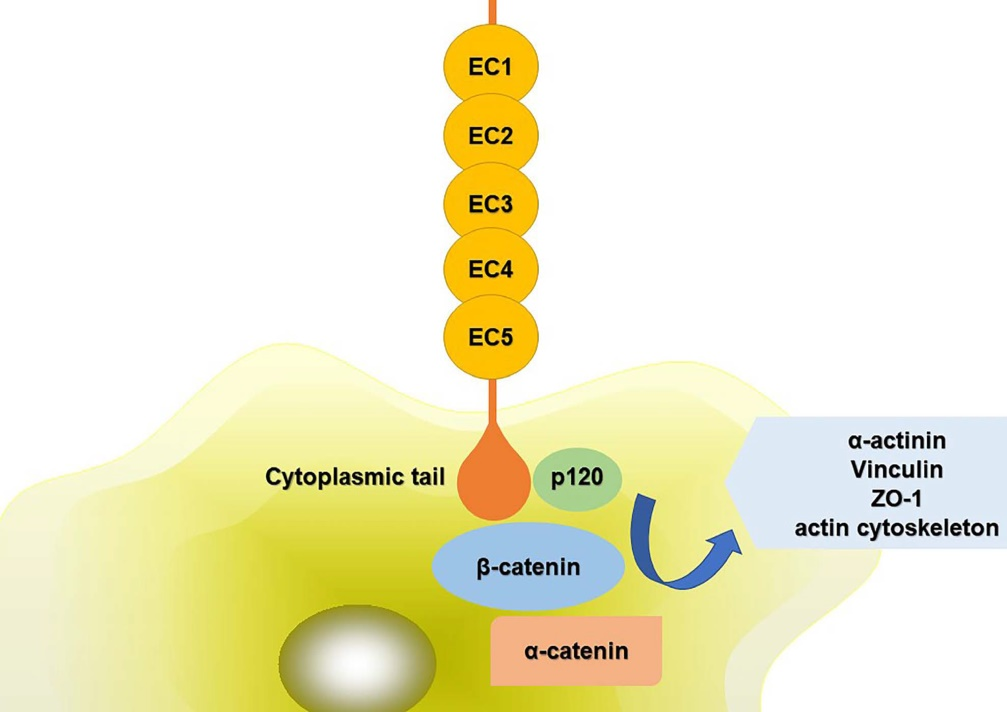
** Schematic diagram of CDH11 mediated cell adhesion.** The extracellular domains of CDH11 have five repeat sequences, called EC1-EC5. The cytoplasmic tail controls the function of CDH11. It can interact with p120ctn, α-catenin, β-catenin to form complexes. And the compound can further interact with α-actinin, vinculin, ZO-1 and the actin cytoskeleton. Abbreviations: ZO-1: zonula occludens-1; EC1-5: five extracellular domains of cadherin-11; p120: p120 catenin.

The extracellular domains of CDH11 have five repeat sequences, called EC1-EC5, and each of them is consisted of about 80-90 amino acid residues. Ca^2+^ can combine with this area to turn the domains into a club-shaped structure, and then initiate the biological effects of cadherin 5,6. The cytoplasmic tail controls the function of CDH11. It can combine with p120^ctn^, α-catenin, β-catenin or plakoglobin (γ-catenin) to form complexes, mediating cell adhesion and regulating signal transduction. The compound can further interact with α-actinin, vinculin, zonula occludens-1 (ZO-1) and the actin cytoskeleton (**Fig. 1**). The phosphorylation of β-catenin and p120^ctn^ leads to the separation of cadherins 7. Alimperti et al. 8 found that CDH11 was necessary to induce the differentiation of MSCs into contractile SMCs. CDH11 regulated the expression of transforming growth factor (TGF)-β1 and affected the differentiation of SMCs through TGF-β receptor II (TGF-β-RII) pathway. At the same time, CDH11 could also activate the expression of serum response factor (SRF) and SMCs protein through Rho-associated protein kinase (ROCK) pathway 9. Cheng et al. 10 found that TGF-β1 increased the expression of CDH11 by activating SMAD2/3-Snail signaling pathway, thus promoting the differentiation of human trophoblast cells. In addition, TGF-β1 could also activate some non-classical signaling pathways such as mitogen-activated protein kinase (MAPK) and PI3K/Akt. Activation of PI3K/Akt signaling pathway increased Snail protein expression, PI3K/Akt/Snail axis could promote the growth, migration and invasion of tumor cells 11. CDH11 extracellular binding domain (CDH11-Fc) could induce the phosphorylation of platelet-derived growth factor receptor (PDGFR)-α fibroblasts and synovial fibroblasts. PDGFR-α-dependent signal stimulated cell proliferation through phosphorylation of PI3K/Akt and MAPK 12.

The gene of CDH11 is situated in 16q22.1, and its complementary DNA (cDNA) is about 3.8 kb, which contains 16 exons and codes 796 amino acids 5. Some studies identified CDH11 as the only 1-Mb hemizygous deletion gene detected at 16q22.1. According to the current epigenetic research, CDH11 has often been inactivated by promoter methylation in a variety of tumors. CDH11 inhibits cell invasion and proliferation, induces tumor cell apoptosis, and finally achieves its anti-cancer effect through Wnt/β-catenin and AKT/Rho A signaling pathways. Methylation-specific PCR (MSP) analysis has also confirmed the frequent methylation of CDH11 in breast cancer, nasopharyngeal carcinoma, esophageal cancer and other cell lines 3.

## The role of CDH11 in different diseases

It has shown that CDH11 plays significant roles in different diseases, and the detailed mechanisms including involved signaling pathways are summarized in **Table 1.**

## Rheumatoid Arthritis (RA) and CDH11

RA is a chronic inflammatory disease, and the main site of inflammation is synovium. In the process of RA, inflammatory synovium tissue experiences severe remodeling and proliferation, fibroblast proliferation, forming an invasive tissue block, called pannus. Pannus tissue can extend to and attach to cartilage, mediate the degradation of cartilage and bone, and then invade these solid tissues 6. Pannus tissue is consisted of a large number of fibroblast-like-synoviocytes (FLS) and macrophages. FLS is a kind of joint mesenchymal cells, which consists of 1-3 cells, forms a membrane between articular cavity and fibrous joint capsule, provides nutrition and lubrication for avascular articular cartilage, and plays an important role in normal and inflammatory synovium 13-15. FLS is the main cell type at the junction of pannus and cartilage erosion, maintaining chronic inflammation which leads to joint destruction 16.

In joints, CDH11 is mainly expressed in FLS. Studies have shown that CDH11 can regulate the migration, invasion and degradation of joint tissue, which mediated by FLS, suggesting that CDH11 expressed by FLS plays significant roles in the etiopathogenesis of RA 5,17-19. Park et al. 20 proved that IL-17 induced the expression of CDH11 in FLS, which could aggravate synovitis and bone destruction. In RA, CDH11 participated in tissue remodeling, led to the aggregation of angiotensin cell clusters, promoted the invasion of angiotensin into articular cartilage, and produced proinflammatory mediators through FLSs in order to enhance the inflammatory response and participate in the inflammatory process of RA 21. CDH11 and its related intracellular molecular complexes can be used as a molecular tool to allow pannus to extend and invade into articular cartilage pathologically 13,14. FLS not only regulates inflammatory response in synovial microenvironment, but also is a target of inflammatory cytokines 22. CDH11 can passively mediate cell adhesion, and bind to the same type of cadherin on adjacent cells through the interaction between its extracellular domains. The recombinant soluble form of CDH11-Fc has a high biological activity. It can stimulate synovial fibroblasts in order to produce chemokines, cytokines and matrix metalloproteinases (MMPs), which work together with inflammatory cytokines to promote the activation of synovial fibroblasts, thus promoting the pathogenesis of RA and cartilage erosion 13,14,23, and studies have shown that estrogen can enhance this inflammatory response 24. The binding of CDH11 on the cell surface to CDH11-Fc can induce the expression of interleukin-6 (IL-6), chemokines and metalloproteinases, which related to the activation of MAPK and nuclear factor-κB (NF-κB) induced by the Fc tail of immunoglobulin. In another study, CDH11 induced IL-6 production through MAPK signaling or NF-κB activation, and synergized with other pro-inflammatory molecules such as tumor necrosis factor-α (TNF-α) and IL-1β to enhance the expression of these inflammatory mediators 22,25. Yoshioka et al. 26 elucidated the role of CDH11 in TNF-α induced RA-FLS proliferation, which might be a β-catenin library. Moreover, Wu et al. 27 found that PI3K/Akt pathway was related to the expression of CDH11 induced by pressure or inflammation, which might be involved in the pathogenesis of arthritis 9.

Thus, CDH11 plays a key role in the progression of RA. Proinflammatory cytokines upregulate CDH11, and then promote FLS homophilic interaction mediated by CDH11, leading to inflammation aggravation and diffusion 22. CDH11 is a potential therapeutic target for RA 28. However, another study showed that CDH11 targeting regimen was not suitable for RA treatment 29.

## Neoplasm and CDH11

### Breast Cancer

#### Invasive breast cancer

In breast cancer, CDH11 can only be expressed in cells and specimens of invasive breast cancer 30, while peripheral CDH11 expression enhances the migration and metastasis of breast cancer cells, indicating that CDH11 plays a key part in the development of breast cancer 31,32. The bone metastasis of breast cancer causes significant incidence rate and mortality. In breast cancer cells, up regulation or overexpression of CDH11 may lead to increased bone metastasis in breast cancer 33.

A specific transcription factor (TCF) of CDH11, Homeobox C8 (HOXC8), activates the transcription of CDH11 by directly combining with the TAA-TCC sequence, which located at nucleotide -196 ~ -191 of the CDH11 promoter 34. Recent studies have found the level of HOXC8 in transitional breast cancer cells significantly increases, suggesting that the presence of HOXC8 may affect the migration of breast cancer cells. CDH11 mediates the regulation of HOXC8 on cell migration. The HOXC8-CDH11 axis promotes the membrane localization of Trio, forming a fresh signal axis HOXC8-CDH11-Trio, which can promote Rac activation in metastatic cancer cells, thus promoting the growth, invasion, migration and metastasis of breast cancer cells 35. Zhang et al. 31 proved that interleukin enhancer-binding factor 3 (ILF3) could combine with CDH11 promoter at nucleotides - 2982 ~ - 2978 and - 2602 ~ 2598, and activate CDH11 transcription by interacting with HOXC8. Further exploring its clinical relevance, it was found that the high expression of HOXC8 and CDH11 was related to the low recurrence free survival rate of breast cancer patients, which further indicated that targeting the HOXC8-CDH11 signal axis was a direction of developing anti metastasis therapy for breast cancer 34,35.

#### Triple Negative Breast Cancer (TNBC)

Among all breast cancer cases, the proportion of TNBC is about 10%-20%. TNBC has special biological behavior and clinicopathological characteristics, so its prognosis is worse than other types.

Based on the analysis of large data of breast cancer, it was found that the expression of CDH11 was positively correlated with the expression of Wnt signal components such as β-catenin, Wnt2 and TCF2, and the expression of CDH11 and β-catenin in TNBC tissues was high at the same time. CDH11 regulated the expression level of β-catenin, and played a key role in enhancing the stem cell, migration and invasion potential of TNBC cells by activating and regulating the typical Wnt-signaling pathway through the CDH11/β-catenin signal axis 36. Chen et al. 37 found that there was a negative correlation between CDH11 and MicroRNA-335 (miR-335), exposure to a single specific anti-CDH11 antibody could remarkably increase the ratio of miR-335/CDH11, inhibit the expression of CDH11, β-catenin and vimentin, and inhibit the metastasis potential.

By targeting β-catenin and CDH11 to regulate the typical Wnt-signaling pathway and inhibit the cancer stem cell (CSC) like phenotype and metastasis phenotype of TNBC cells, it represents a new method of TNBC treatment, which provides a basis for further exploring CDH11 as a candidate target of TNBC targeted treatment 36,37.

### Colorectal cancer (CRC)

CRC is one of the most common cancers 38. Its specific pathogenesis includes the activation of oncogenes and the inactivation of tumor suppressor genes.

Microcystin-LR (MC-LR) is a kind of tumor initiator, which has been confirmed to participate in the occurrence of human primary cancer. The results of PCR displayed that the expression of CDH11 gene increased significantly after MC-LR exposure, while the cell migration and invasion induced by CDH11 gene knockout decreased, suggesting that MC-LR upregulated the expression of CDH11 in HT-29 cells, and enhanced the cell migration and invasion. Therefore, CDH11 is a tumor promoter, which participates in the cytoskeleton reorganization and cell movement induced by MC-LR, so as to enhance the motility and invasiveness of cancer cells. It is suggested that the inhibition of CDH11 expression may be a new and promising therapeutic strategy for CRC 39.

The inactivation of tumor suppressor genes is closely related to epigenetic changes. DNA of tumor cell lines copy number distortion and 1-Mb hemizygote deletion at 16q21-22.1, and CDH11 is the sole known gene situated in the deletion, so CDH11 may be a candidate tumor suppressor gene related to 16q21-22.1 deletion 3. In CRC, the down-regulation of CDH11 is caused by promoter methylation, and the level of CDH11 methylation is particularly high in CRC tissues. CDH11 can induce cell periodic arrest and apoptosis in G0 or G1 phase, inhibit the proliferation of CRC cells and formation of colonies, and inhibit tumor cell growth, migration, invasion and proliferation by inactivating NF-κB signal pathway. Therefore, CDH11 is considered as a functional tumor suppressor gene of CRC, which can be used as a prognostic biomarker of value for further study 38.

### Prostate cancer (PCa)

PCa has been one of common cancers in humans in recent years. Since the proliferation of PCa cells is stimulated by androgen receptor (AR), the main method to treat the advanced PCa now is androgen ablation. However, some patients treated with androgen deprivation therapy eventually became resistant to it and developed bone metastases 40. Bone metastasis is the leading cause of PCa-related death 41. It was found that CDH11 was highly expressed in the PC-3 cell line in bone metastases. In addition, when the CDH11 gene was specifically knocked out, the incidence of PC-3 bone metastasis was significantly reduced, providing evidence for the correlation between CDH11 and PCa 42. Satcher et al. 43 confirmed that clathrin mediated the endocytosis of CDH11 through a unique structural motif, the “clathrin-binding motif” VFEEE, thus regulating the migration of PCa cells mediated by CDH11. Another study further demonstrated that CDH11 mediated the adhesion of PCa cells to osteoblasts, enabling PCa cells to insert into osteoblasts and promoting the proliferation, invasion and migration of PCa cells. Furthermore, according to microarray analysis, since CDH11-mediated cell invasion and migration were rely on its cytoplasmic domain, it suggested that CDH11 might activate a signaling pathway that promoted the migration of PCa cells to bone 44.

Since bone metastasis often occurs after the progression of castration resistance, studies have shown that androgen depletion can lead to the up-regulation of CDH11, thereby increasing the metastasis of PCa to bone. In other words, treatment strategies that blocked the expression or function of CDH11 may reduce the possibility of bone metastasis in patients undergoing androgen ablation, which provides an idea for clinical treatment 40. For example, the dimer of CDH11 extracellular domain was expressed as Fc fusion protein (OB-CAD-FC), which can competitively inhibit the combination of osteoblasts and PCa cells 45. Some studies have focused on the monoclonal antibodies against the extracellular domain of CDH11, targeting two kinds of antibodies, mAb 2C7 and 1A5, which have been proved to be able to significantly reduce bone metastasis *in vivo*, but the specific mechanism still needs further study 46.

### Other cancers

In addition to these cancers, there are many other cancers that are associated with CDH11.

Consistent with the possibility that CDH11 may participate in bone metastasis in breast cancer and PCa in former researches, studies have indicated that CDH11 is involved in bone organ-specific metastasis of 786-O cell lines by increasing the migration of renal cell carcinoma (RCC) cells or the adhesion of RCC to osteoblasts in bone marrow 47. Carmona et al. 48 proved that CDH11 also played an important role in the early diagnosis of RCC, and CDH11 gene might be a cancer suppressor gene during the initiation and development of RCC. CDH11 gene methylation was associated with tumor invasion and metastasis, suggesting that deletion of CDH11 gene expression caused by DNA methylation was involved in the occurrence and development of a variety of tumors. Therefore, CDH11 gene expression can be induced by demethylation drugs to treat tumors.

CDH11 is also involved in the activation of pancreatic stellate cells (PSCs) and migration of pancreatic cancer cells. CDH11 plays an inflammation-inducing role in EMT and is involved in progression from chronic pancreatitis to pancreatic cancer 3,49. The study detected that CDH11 might be involved in the feedforward mechanism mediating TGF-β generation and signal transduction. The expression level of CDH11 was up-regulated in diseased pancreatic tissues, and maintained PSCs activated phenotype 50,51. The synthesis of TGF-β and the continuous activation of PSCs led to the over synthesis of extracellular matrix (ECM) and promoted pancreatic fibrosis and metastasis of pancreatic cancer cells 52.

In addition, Lin et al. 53 found that hypermethylation of CDH11 abnormal promoter in bladder cancer (BCA) cells led to the epigenetic inactivation of CDH11, and BCA with high CDH11 expression had poor prognosis 54. Zhang et al. 55 found that the bidirectional regulation of C12orf59 and CDH11 formed positive feedback, promoted the growth of Gastric cancer (GC) cells, and maintained the metastasis and invasion of human GC cells. Eyvazi et al. 56 also proved promoter CpG island hypermethylation of CDH11 gene in patients with GC. Studies have shown that the migration of lung cancer cells and head and neck cancer cells can be inhibited by regulating the adhesion of ECM and the expression of CDH11 57,58. In addition, CDH11 is one of the potential biomarkers of diffuse large B-cell lymphoma (DLBCL) and plays an important role in predicting central nervous system recurrence 59. CDH11 is also expressed in stromal cells of ovarian tumors, such as myofibroblasts, vascular SMCs, and endothelial cells 17.

## Calcific Aortic Valve Disease (CAVD) and CDH11

CAVD can cause aortic thickening and hardening, which impairs the fine regulation function and eventually leads to regurgitation and stenosis. The risk of heart disease, atherosclerosis, heart failure and stroke will be increased in patients with early-onset CAVD 60. Because there are no effective biomarkers to diagnose or delay the disease progression, it is very important to identify the signal pathways that mediate the homeostasis and initiate the disease progression in the valve for the development of potential diagnostic and therapeutic targets 61.

Myofibroblasts play important parts in the development process of CAVD. TGF-β1 can induce myofibroblast differentiation of aortic valve interstitial cells (AVICs), resulting in the up regulation of α-smooth muscle actin (α-SMA) and CDH11 in an ERK1/2-dependent manner, as well as an increase in contractility, cell-cell connectivity and intercellular tension. Finally, the tension of the whole monolayer cells increases, resulting in the imbalance of production force, which leads to the apoptosis of weak cells and the formation of aggregates as the initial step of morphogenesis of calcified nodules. When the calcified nodules mature, the cells around the aggregates underwent strain amplification due to the non-deformation of the nodules, leading to apoptosis and calcification 62-65.

The mechanism of CDH11 participating in valve calcification and ECM reconstruction in adults is accomplished by RhoA/Sox9 *in vivo* and *in vitro*. RhoA and Sox9 are downstream targets of CDH11. Among them, CDH11 increases the sensitivity of cells to mechanical tension through RhoA signal and promotes the differentiation of stromal cells into myofibroblasts and osteoblasts. Up regulation of RhoA and Sox9 leads to excessive mechanical activation and ECM remodeling, leading to morphogenesis that severely impairs valve function. Sox9 can be used as a therapeutic marker to reduce the expression of osteogenic molecules 61. In addition, studies have found that the lack or knockout of CDH11 can destroy the polarity of valve cells, the formation, migration and matrix compaction of stress fibers, reduce RhoA activity mediated filamentous protrusion 66, prevent valve stenosis, lobular thickening and hardening, and thus prevent CAVD. Clark et al. 67 found that CDH11 caused aortic valve stenosis in Notch1 mutant mice, and blocking CDH11 could prevent valve stenosis, lobular thickening and hardening, and the expression of inflammatory genes. Therefore, targeting CDH11 is a new therapeutic strategy to prevent disease progression in patients with hereditary and idiopathic CAVD.

## Skin diseases and CDH11

Scleroderma (systemic sclerosis, SSc) is a kind of connective tissue disease with multiple system damage, with progressive fibrosis of viscera and skin. The complex etiopathogenesis of SSc involves inflammation and autoimmunity, vascular disease, and excessive deposition of ECM 68. Dendritic cells, macrophages and T cells are involved in the activation of fibroblasts and myofibroblasts. Stimulated by TGF-β, they produce a large number of ECM and lead to tissue fibrosis. SSc involves a variety of pathways, such as Type Ⅰ interferon (IFN-I), TGF-β, Wnt-β catenin and CDH11 69.

CDH11 can induce macrophages to produce TGF-β and regulate the level of TGF-β *in vivo*, thus indirectly regulating the behavior of fibroblasts. It is a complex process during the progression of tissue fibrosis in SSc patients, which involves many molecules in a variety of cell types. Early endothelial dysfunction, as well as the activation and recruitment of inflammatory cells (including macrophages and CD4^ +^ T cells) to the skin can lead to the increase of inflammatory cytokines (including CCL2, IL-6 and IFN-I) and pro fibrogenic cytokines (such as interleukin-13 (IL-13) and TGF-β), thus activating tissue injury and wound healing pathways. Under the action of TGF-β and other fibrogenic mediators, fibroblasts aggregate in the fibrotic tissue, resulting in mounting deposition of ECM and tissue fibrosis 70,71.

In conclusion, it has been confirmed that CDH11 is a mediator of skin fibrosis, and anti CDH11 antibody can prevent the development of early inflammatory stage of skin diseases. In addition, CDH11 antagonist can accelerate the resolution of existing fibrosis, suggesting that CDH11 may be a potential therapeutic target for SSc 70.

## Fibrotic diseases and CDH11

There are no reliable diagnostic biomarkers and effective therapeutic drugs for liver fibrosis, however, more and more studies have been focused on the effect of CDH11 in the pathogenesis of liver fibrosis 72-74. Liver fibrosis is accompanied with massive deposition of ECM proteins, which are mainly from activated hepatic stellate cells (HSCs). Numerous studies have shown that CDH11 is expressed on a variety of cells in liver fibrosis, including macrophages, injured hepatocytes and HSCs. CDH11 regulates TGF-β production in macrophages, induces HSCs activation, transdifferentiates into highly proliferative myofibroblasts, and secretes a variety of ECM proteins. Excessive ECM destroys normal liver structure, leading to cirrhosis 74,75.

In addition, CDH11 can also mediate the contact with fibroblasts, and promote pulmonary fibrosis by regulating the production of TGF-β in alveolar macrophages and EMT in alveolar epithelial cells 71,76,77. CDH11 even participates in the development of intestinal fibrosis and renal fibrosis 78, suggesting that CDH11 is a common mediator of fibrosis in various tissues, as well as indicating that targeting CDH11 used in the treatment of fibrosis diseases is worthy of further study 75.

## Other diseases and CDH11

Adipose tissue inflammation is a metabolic disease that drives the development of insulin resistance and diabetes in obese patients 79. Many cells contribute to the control of adipose tissue inflammation, such as M2 macrophages, eosinophils, Tregs, innate lymphoid type 2 cells (ILC2s), and invariant NKT (iNKT) cells. The study found that the production of PDGFR^α+^ fibroblasts of interleukin-33 (IL-33) was increased in CDH11 deficient mice, while the proliferation of M2 macrophages, ILC2s and IL-13 was enhanced. IL-33 induced the activation and proliferation of ILC2s to express IL-13 and interleukin-5 (IL-5), which induced M2 macrophage and eosinophil aggregation, as well as regulated Treg homeostasis of adipose tissue, contributing to the control of adipose tissue inflammation. Experiments have also demonstrated that CDH11 deficiency significantly prevents adipose tissue inflammation and metabolic syndrome in obese mice 80,81. Therefore, CDH11 targeting is of great significance in the treatment of adipose tissue inflammation, diabetes and metabolic syndrome.

Myocardial infarction (MI) can remarkably reduce cardiac function and may exacerbate the development of heart failure. The process of infarct healing begins with the involvement of resident cells and newly recruited cells in healing, remodeling, and cardiac stabilization, followed by coordinated clearance and replacement of injured tissue by immune cells to form a dense, robust and stable collagen scar that maintains the integrity of the myocardial wall to prevent cardiac rupture 82. Studies have shown that cells expressed by CDH11 lead to inflammation-driven fibrotic remodeling after MI through regulating the recruitment of bone marrow-derived cells, then limiting fibroblast-induced IL-6 expression and promoting vascularization, thereby improving prognosis. It suggests that CDH11 plays an important part in solving tissue rupture and promoting myocardial remodeling. CDH11 may serve as a potential therapeutic target, so targeting CDH11 may reduce early inflammation and tissue breakdown mediated by neutrophil and macrophage, later collagen deposition mediated by myofibroblast and inflammation and fibrotic remodeling after MI 83.

## Conclusion and Perspectives

At present, DNA demethylation is an important way to restore tumor suppressor gene function 84. CDH11 can inactivate promoter methylation. Therefore, increasing researchers have carried out related research on CDH11 targeted disease treatment. They explored the targeted therapy of CDH11 by controlling gene knockout and screening anti-CDH11 antibody. In cancers, previous studies have shown that knockout of CDH11 gene can reduce the size of the formed mammary bulb and inhibit breast cancer tumorigenesis 36. Chen et al. 37 treated human metastatic breast cancer cell lines MCF7 and MDA-MB-231 with a single specific anti-CDH11 antibody mediated by miR-335, which proved that the antibody reduced the CD44^hi^CD24^neg/lo^ cell population, inhibited the motility of breast cancer cells and the efficiency of mammary bulb formation. Lee et al. 46 studied two kinds of antibodies, mAb 2C7 and 1A5, which blocked CDH11 mediated cell adhesion, and determined that the 343-348 motif of EC3 could be used as a good target for the treatment of CDH11 antibody. It was proved that they can significantly reduce bone metastasis *in vivo* and achieve the treatment of PCa. In CAVD, Clark et al. 67 found that targeting CDH11 could prevent the disease progression of hereditary CAVD caused by Notch1 mutation. In skin diseases, some studies have shown that anti-CDH11 monoclonal antibody can inhibit CDH11, and regulate TGF-β level *in vivo* and TGF-β production by macrophages, which can effectively reduce skin fibrosis and accelerate the resolution of existing fibrosis 70,71. In addition, there are many studies on the role of blocking CDH11 in RA and metabolic diseases.

CDH11, as a member of the cadherin superfamily, is a group of transmembrane proteins that are mainly located at the junction of adhesion molecules and mediate homophilic intercellular adhesion 51. It can regulate multiple aspects of cell behavior, including proliferation, differentiation, apoptosis, cell polarity, self-renewal and differentiation of embryonic stem cells, as well as maintenance of tissue integrity 1, which plays important roles in multiple cellular tissues. This review focuses on the progress of research on the role and mechanism of CDH11 in the development of various diseases, and discovers some rules of the role of CDH11. In conclusion, CDH11 may be an effective target for the treatment and prevention of various diseases, such as arthritis, cancer, aortic calcification disease, dermatosis, etc. Therefore, CDH11 targeted therapy will become a promising therapeutic strategy. Nowadays, there have been a lot of research results on CDH11 as a target therapy for various diseases, but the specific mechanism of CDH11 targeted therapy needs to be further studied and clarified. More research work is still needed to understand the mechanism of CDH11 acting on these diseases and reveal the molecular pathways guiding these processes, which is of great significance for discovering new therapeutic ideas and methods.

## Figures and Tables

**Table 1:** The mechanism of CDH11 in different diseases

Diseases	Mechanism	Signaling pathways	Reference
RA	angiotensin↑ FLS↑		21
	CDH11-Fc↑ MMPs↑	PI3K/Akt	9,13,14,23, 27
	IL-6↑ TNF-α↑ IL-1β↑	MAPK or NF-κB	22,25
Invasive Breast Cancer	ILF3↑ HOXC8↑	HOXC8-CDH11-Trio	31,34,35
TNBC	β-catenin↑ Wnt2↑ TCF2↑ miR-335↓	Wnt/β-catenin	36,37
CRC	MC-LR↑	NF-κB	38,39
PCa	VFEEE	Intracellular signal pathway	43,44
RCC	DNA methylation		48
Pancreatic cancer	EMT↑ TGF-β↑ ECM↑		3,49-52
BCA	abnormal hypermethylation epigenetic inactivation of CDH11		53,54
GC	promoter CpG island hypermethylation		55,56
Lung cancer	ECM↑		57
Head and neck cancer	ECM↑		58
CAVD	TGF-β1↑ α-SMA↑	ERK1/2-dependent	62-65
	ECM reconstruction	RhoA/Sox9	61
SSc	TGF-β↑ IFN-I↑ ECM↑	Wnt/β-catenin	69,70,71
Liver fibrosis	TGF-β↑ ECM↑		74,75
Pulmonary fibrosis	TGF-β↑ EMT↑		71,76,77
Adipose tissue inflammation	CDH11 deficient: PDGFR^α+^↑ ILC2s↑ IL-13↑ M2 macrophages↑		80,81
MI	IL-6↑		83

Abbreviations:CDH11:Cadherin-11,OB-cadherin; EMT: epithelial-mesenchymal transition; RA: Rheumatoid arthritis; FLS: fibroblast-like synoviocytes; CDH11-Fc: CDH11 extracellular binding domain; MMPs: matrix metalloproteinases; TNF-α: tumor necrosis factor-α; IL-6: Interleukin-6; IL-13: Interleukin-13; MAPK: mitogen-activated protein kinase; HOXC8: Homeobox C8; ILF3: interleukin enhancer-binding factor 3; TNBC: Triple negative breast cancer; TCF: transcription factor; miR-335: MicroRNA-335; CRC: Colorectal cancer; MC-LR: Microcystin-LR; NF-κB: nuclear factor-κB; PCa: Prostate cancer; RCC: renal cell carcinoma; TGF: Transforming growth factor; BCA: bladder cancer; GC: Gastric cancer; CAVD: Calcified aortic valve disease;α-SMA: α- smooth muscle actin; ECM: extracellular matrix; SSc: Scleroderma, systemic sclerosis; IFN-I: type I interferon; PDGFR: platelet-derived growth factor receptor; ILC2s: lymphoid type 2 cells; MI: Myocardial infarction.

## Abbreviations

CDH11: Cadherin-11, OB-cadherin
CDH1: Cadherin-1, E-cadherin
CDH3: Cadherin-3, P-cadherin
CDH5: Cadherin-5, VE-cadherin
CDH8: Cadherin-8
CDH13: Cadherin-13, H-cadherin
SMCs: smooth muscle cells
EMT: epithelial-mesenchymal transition
MSCs: mesenchymal stem cells
ZO-1: zonula occludens-1
SRF: serum response factor
ROCK: Rho-associated protein kinase
MAPK: mitogen-activated protein kinase
cDNA: complementary DNA
MSP: methylation-specific PCR
RA: Rheumatoid arthritis
FLS: fibroblast-like synoviocytes
CDH11-Fc: CDH11 extracellular binding domain
MMPs: matrix metalloproteinases
TNF-α: tumor necrosis factor-α
IL-6: Interleukin-6
IL-13: Interleukin-13
IL-33: Interleukin-33
IL-5: Interleukin-5
HOXC8: Homeobox C8
ILF3: interleukin enhancer-binding factor 3
TNBC: Triple negative breast cancer
TCF: transcription factor
miR-335: MicroRNA-335
CSC: cancer stem cell
CRC: Colorectal cancer
MC-LR: Microcystin-LR
PCR: polymerase chain reaction
NF-κB: nuclear factor-κB
PCa: Prostate cancer
AR: androgen receptor
OB-CAD-FC: Fc fusion protein
RCC: renal cell carcinoma
PSCs: pancreatic stellate cells
TGF: Transforming growth factor
BCA: bladder cancer
GC: Gastric cancer
DLBCL: diffuse large B-cell lymphoma
CAVD: Calcified aortic valve disease
AVICs: aortic valve interstitial cells
α-SMA: α-smooth muscle actin
ECM: extracellular matrix
SSc: Scleroderma, systemic sclerosis
IFN-I: type I interferon
HSCs: hepatic stellate cells
PDGFR: platelet-derived growth factor receptor
ILC2s: lymphoid type 2 cells
iNKT: invariant NKT cells
MI: Myocardial infarction
